# Recruitment, retention and reporting of variables related to ethnic diversity in randomised controlled trials: an umbrella review

**DOI:** 10.1136/bmjopen-2024-084889

**Published:** 2024-08-09

**Authors:** Ebenezer Owusu-Addo, Deborah M Bennor, Aaron Michael Orkin, An-Wen Chan, Vivian A Welch, Shaun Treweek, Heidi Green, Peter Feldman, Davina Ghersi, Bianca Brijnath, On behalf of the RECONSIDER Extension Group, Hayat Ahmed, Nita Bhandari, Barbara E Bierer, Owen Chinembiri, Kenzie Cameron, Daniel Coase, Maria Cuervas, Shoba Dawson, Robert Golub, Farrokh Habibzadeh, Merilyn Heuschkel, Lindsey Jasicki, Lillian Leigh, Tianjing Li, Lawrence Mbuagbaw, Raylynn Benn, John Norrie, Mayra Ouriques, George Papadopolous, Dawn Richards, Nandi Siegfried, Nicola Straiton, Jvan Yazdani, John Zalcberg

**Affiliations:** 1 Bureau of Integrated Rural Development, Kwame Nkrumah University of Science and Technology, Kumasi, Ashanti, Ghana; 2 Department of Family and Community Medicine and Dalla Lana School of Public Health, University of Toronto, Toronto, Ontario, Canada; 3 Women’s College Research Institute and Department of Medicine, University of Toronto, Toronto, Ontario, Canada; 4 Methods Centre, Bruyere Research Institute, Ottawa, Ontario, Canada; 5 Campbell Collaboration, University of Ottawa Faculty of Medicine, Ottawa, Ontario, Canada; 6 Health Services Research Unit, University of Aberdeen, Aberdeen, Scotland, UK; 7 COUCH Health, Manchester, UK; 8 Social Gerontology, National Ageing Research Institute Inc, Parkville, Victoria, Australia; 9 School of Public Health and Preventive Medicine, Monash University, Melbourne, Victoria, Australia; 10 The University of Melbourne - Parkville Campus, Melbourne, Victoria, Australia

**Keywords:** Clinical Trial, Health Equity, Review

## Abstract

**ABSTRACT:**

**Objective:**

This umbrella review synthesises evidence on the methods used to recruit and retain ethnically diverse participants and report and analyse variables related to ethnic diversity in randomised controlled trials.

**Design:**

Umbrella review.

**Data sources:**

Ovid MEDLINE, Ovid Embase, CINAHL, PsycINFO and Cochrane and Campbell Libraries for review papers published between 1 January 2010 and 13 May 2024.

**Eligibility criteria:**

English language systematic reviews focusing on inclusion and reporting of ethnicity variables. Methodological quality was assessed using the AMSTAR 2 tool.

**Results:**

Sixty-two systematic reviews were included. Findings point to limited representation and reporting of ethnic diversity in trials. Recruitment strategies commonly reported by the reviews were community engagement, advertisement, face-to-face recruitment, cultural targeting, clinical referral, community presentation, use of technology, incentives and research partnership with communities. Retention strategies highlighted by the reviews included frequent follow-ups on participants to check how they are doing in the study, provision of incentives, use of tailored approaches and culturally appropriate interventions. The findings point to a limited focus on the analysis of variables relevant to ethnic diversity in trials even when they are reported in trials.

**Conclusion:**

Significant improvements are required in enhancing the recruitment and retention of ethnically diverse participants in trials as well as analysis and reporting of variables relating to diversity in clinical trials.

**PROSPERO registration number:**

CRD42022325241.

**STRENGTHS AND LIMITATIONS OF THIS STUDY**

- Comprehensive evidence synthesis of trials across medical research to identify ways to improve the representation of ethnic diversity in trials.
- Identifies gaps in the literature on recruitment and retention of ethnic diversity in trials and provides recommendations for the future.
- The primary studies in the included reviews were predominantly from the USA.

## Introduction

 Randomised controlled trials (hereafter, trials) are considered the gold standard method to assess the effectiveness and efficacy of healthcare interventions.^1^ Their results directly inform much of clinical practice, particularly through clinical guidelines. Failure to include participants who are representative of a population’s true ethnic diversity could render trial results not fully generalisable to the affected population and that may not address the needs, values and experiences of diverse communities.^2 3^ Further, the exclusion and underrepresentation of ethnic diversity in trials undermine efforts to reduce health disparities and facilitate health equity. Recruiting a diverse and representative population into trials and reporting on sociodemographic characteristics of study populations, including ethnicity, are therefore important to support the validity and generalisability of trials and are important for health equity and health justice.^4^

However, this goal is not without challenges, as ethnicity represents multiple intersecting factors that are geographically, linguistically and contextually specific and localised. Terminology, variables and related disclosures that may be appropriate or even desirable in one jurisdiction or context may be misinterpreted, inappropriate or illegal in another. This inhibits the development of universal or standardised variables to represent ethnicity in study populations, and researchers report difficulty in identifying which variables to collect, how to report them and how to conduct appropriate analyses using those variables.^5 6^

While reviews on consideration of ethnicity in guidelines and systematic reviews do exist, most have had either a disease-specific focus (eg, dementia,^7^ mental health,^8^ dermatology^9^) and/or catalogue the extent of underrepresentation of ethnic diversity in trials but offer few solutions to overcome known barriers. This makes it difficult to glean clear and coherent guidance that researchers can use to inform how they collect and report on ethnic diversity. With the increase in the number of reviews focused on consideration of ethnicity in trials, a fundamental next step is to provide researchers, patients, policymakers, clinicians and public health practitioners the evidence they require to conduct more inclusive medical research. A review of reviews provides a coherent appraisal and summary of reviews, allowing the findings of individual reviews to be compared and contrasted,^10 11^ thus facilitating the development of good practice guidelines. This systematic review of reviews will provide a valuable overview of the current state of evidence on ethnicity reporting. We hope that this review will set the stage for guidelines that can help address this area of trial methodology and health equity.

## Review aim

This review of reviews synthesises evidence on the methods used to recruit and retain participants from diverse ethnic backgrounds in trials and report and analyse variables related to ethnicity in trials.

The review is guided by the following questions:

1. What methods are used to recruit and retain participants from ethnically diverse backgrounds?
2. What variables related to ethnicity are collected and reported?
3. What types of analyses are conducted with ethnic diversity variables and how are differences between ethnic groups accounted for?

## Methods

### Conceptualising ethnicity

The language and terminology used to describe ethnicity and ethnic groups can be sensitive, due, in part, to the close association between the identity; the history and legacy of colonisation, violence and trauma; societal and structural racism; and contemporary settings and norms. Historically, it has been established that mainstream and state-sponsored medicine, health agencies and health research have played various leading, active and complicit roles in building and entrenching ethnic inequities and injustices.^12 13^

In this article, we recognise that both ‘race’ and ‘ethnicity’ are social constructs and not biological categories. Governments and institutions around the world tend to make use of one or both terms (or, when not in English, similar notions).^14^ However, the concept of ‘race’ is controversial. The biological concept of race, whereby human populations are divided into sub-categories mainly based on visible physical characteristics, was dominant from the early 19th century. Modern ideas of race focus on social origins rather than biology. Even in this conception, race is based on visible physical features and is therefore perceived as biologically bound. We therefore avoid the term ‘race’ in our analysis but use it in reference to those studies where it is listed as such (even if they are not our preferred terms).^14^

For our analysis, we focus on ethnicity, recognising that, even here, the nomenclature is varied and not without problems. For example, in Australia, the term ‘Culturally and Linguistically Diverse’ is used, while in the UK, the term ‘Black And Minority Ethnic’ was in use pre-pandemic but has been replaced with ‘ethnic minorities’ or ‘global majority’ in recent years. In the USA, the term ‘minority ethnic’ or ‘ethnically minoritised’ applies, and in parts of Europe, ethnically diverse communities are often categorised as ‘immigrants’ and/or of immigrant background, irrespective of whether they are first or subsequent generations of migrants. What these terms have in common is that, overall, they signify a group of people who are currently not of the majority population in their settlement country. However, with population diversity, migration and growing global interconnections, this trend is likely to change. We also acknowledge the complex and intersecting relationships between ethnicity, culture, religion and minority status. We further acknowledge that marginalised ethnic groups may not always represent a minority population, especially in colonial contexts (eg, South Africa) and that ethno-racial identities do not always represent visible differences. Therefore, we use the term ‘ethnic diversity’ or variations of the same, cognisant of the inadequacies of the term.

### Patient and public involvement

In line with the GRIPP2 reporting checklists,^15^ this review involved both patients and the public at various stages of the review. The RECONSIDER Extension Group, which includes four ethnically diverse consumers from Australia, the UK and Chile, and trialists and commissioners of research provided useful input in refining the review questions and the search strategy. Findings from the current work will be disseminated through open-access publication at The BMJ; targeted outreach to researchers, patients, policymakers, clinicians, and public health practitioners; social media posts; and potential press releases via colleagues in journalism and science communication. Both patients and the public will be involved in the dissemination of the study results.

### Design

This review is reported in accordance with the Preferred Reporting Items for Systematic Reviews and Meta-Analyses (PRISMA) statement.^16^ We followed a ‘review of reviews’ methodology^17^ focusing on synthesising evidence from related reviews on inclusion and reporting of ethnicity variables, to help inform decisions about different approaches. To improve the internal validity of the review, the methodology for conducting this review of reviews followed recognised guidelines for conducting a systematic review of reviews.^11 18^ The review protocol was registered in PROSPERO (registration number: CRD42022325241).

### Eligibility criteria

Table 1 indicates the review eligibility criteria.

**Table 1:** Eligibility criteria

Inclusion criteria	Explanation
Population	Ethnic minorities, minority racial/ethnic/cultural/linguistic.
Concept	Variables relating to ethnicity in trialsRecruitment: identifying or sourcing participants who may be eligible for the trial. We included trials that described the recruitment of participants from diverse ethnic backgrounds and/or strategies used to enrol participants from diverse ethnic backgrounds in trials.Retention: keeping enrolled participants in a trial from discontinuing or dropping out. We included reviews that described strategies used to keep participants from diverse ethnic backgrounds enrolled in trials.Analysis: analysis of variables operationalising ethnic diversity included in a trial. We included reviews that focused on an analysis of ethnic diversity variables in trials.Reporting: we included reviews that focused on reporting or representation of ethnic diversity in trials.
Type of study	Systematic reviews, meta-analysis, realist reviews or scoping reviews with methods section with explicit inclusion criteria.
Outcomes	Reviews reporting on any of the following: ethnicity-related variables collected; measures of ethnicity created; ethnicity-specific tools used; analyses performed on ethnicity-related variables; type of data analysed (eg, categorical/continuous); corrections, adjustments and sub-group analyses conducted; whether there was a triangulation of measures of ethnicity; techniques used to facilitate recruitment and participation of participants; and techniques used to enhance retention of participants from diverse ethnic backgrounds.
Year of publication	Reviews published between 1 January 2010 and 13 May 2024.
Exclusion criteria	Primary studies and reviews describing case studies or series or descriptive studies only.
	No data on any of the primary outcomes that are of interest to the review.
	Studies not published in English.

### Search strategy

A systematic search was performed in the following databases: Ovid MEDLINE, Ovid Embase, CINAHL, PsycINFO, Cochrane Database of Systematic Reviews, Database of Abstracts of Reviews of Effectiveness (DARE), 3ie Systematic Reviews, Evidence for Policy and Practice Information and Co‐ordinating Centre (EPPI‐Centre) Evidence Library and Campbell Library. Full-text search terms were developed first for MEDLINE and adapted for other databases as necessary. Forward citation searching and manual searching of the reference lists of all included systematic reviews were conducted to minimise the risk of missing relevant studies. This umbrella review was conducted to inform the development of guidelines on the inclusion and reporting of ethnicity in trials (RECONSIDER Extension). Therefore, to ensure that the guideline is up to date, the search was limited to 14 years and included reviews published during the timeframe of 1 January 2010−13 May 2024. EndNote and Covidence were used to manage the search results including the removal of duplicates.

### Selection of studies and data extraction

Two independent reviewers carried out title and abstract screening (EOA and DMB), and full-text screening using Covidence systematic review software. A sample set of 15 papers was used to train the reviewers and establish a shared understanding of eligibility. We conducted independent and duplicate title and abstract screening, full-text review and data extraction. Discordance between reviewers was resolved by consensus and in consultation with a third reviewer (BB/PF) as necessary.

The main components of the data extraction are a review of authors and years of publication; review methodology: aim and research questions, review design and design of its included studies, number of primary studies included, (number of databases searched and date range of databases searched, quality assessment characteristics and ratings); characteristics of included reviews (number of included participants, type of participants and countries/regions); main results/findings: ethnicity-related variables collected; measures of ethnicity created; ethnicity-specific tools used; analyses performed on ethnicity-related variables; type of data analysed (eg, categorical/continuous); corrections, adjustments and sub-group analyses conducted; whether there was triangulation of measures of ethnicity; techniques used to facilitate recruitment and participation of participants; and techniques used to enhance retention of participants from diverse ethnic backgrounds.

### Quality assessment

We conducted independent and duplicate quality appraisals of included studies using the validated AMSTAR 2 tool.^19^ Use of the AMSTAR 2 tool was piloted by two reviewers to establish consistency in agreement before an independent assessment.

### Data synthesis and analysis

To accommodate the extensive variation in how ethnicity in health research is conceptualised, a narrative synthesis^20^ was used for reporting. For each included review, details about the nature and type of ethnicity-related variables, specific elements of ethnicity-related data collection methods and analysis methods were reported by two independent reviewers and verified by a third reviewer. The data points were derived via consensus, drawing on the review team’s knowledge of how variables on ethnicity are operationalised in government-funded statistics agencies and the medical literature.^21^,^23^ Following the Cochrane Handbook for Systematic Reviews of Interventions,^24^ summary tables were used to present the review findings within the framework of a narrative synthesis.

## Results

### Search results

**Figure 1:**
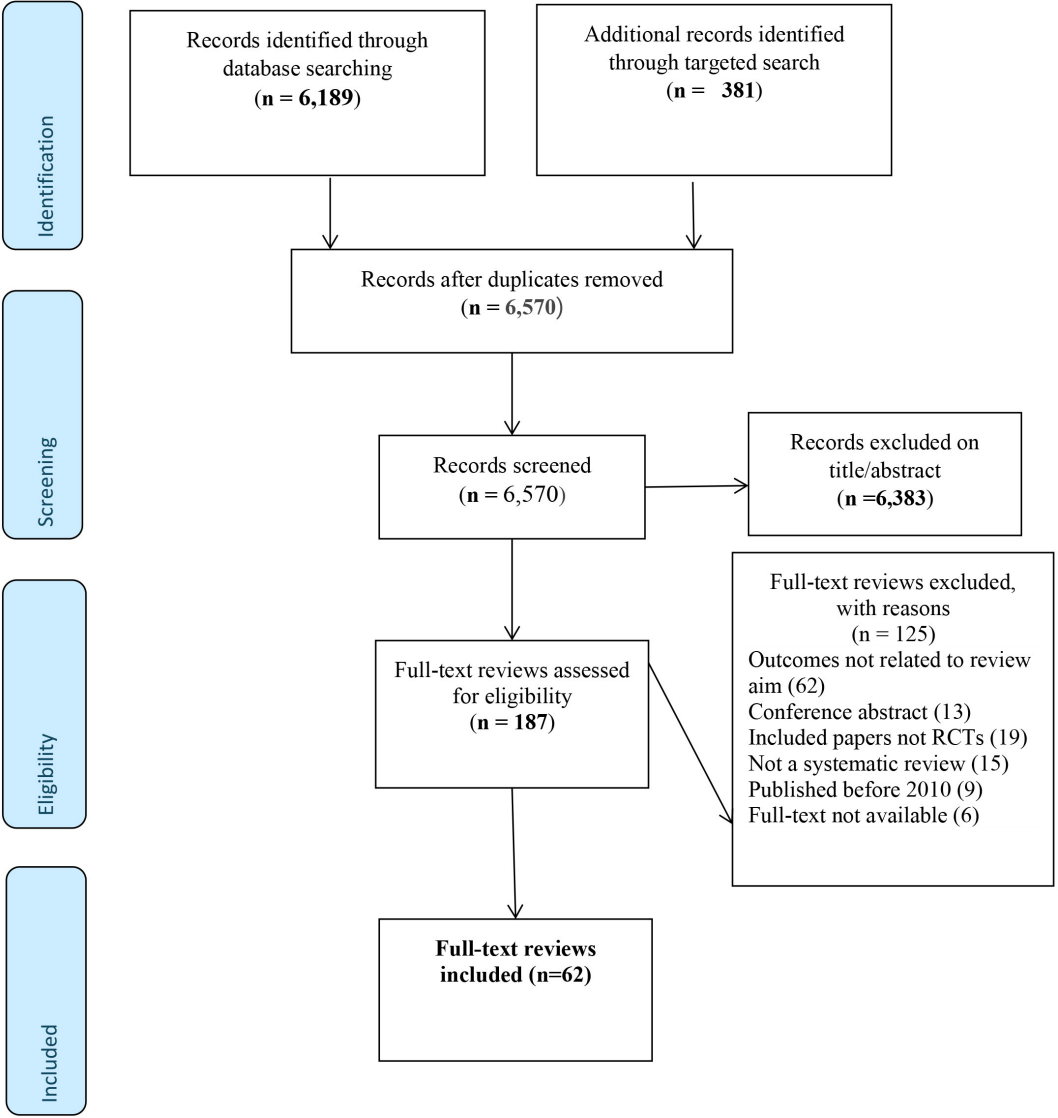
Preferred Reporting Items for Systematic Reviews and Meta-Analyses flow chart.

The PRISMA flow chart (figure 1) shows the review’s selection process. The search yielded 6189 citations, of which 187 full-text reviews were screened for eligibility. Sixty-two full-text systematic reviews met the inclusion criteria for data extraction. We focus here on the results of 62 primary reviews included in the overview.

### Description of included reviews

A summary of the characteristics of the included reviews is presented in online supplement additional file 1. Of the 62 included reviews, 53 were systematic reviews,^925^,^76^ eight were systematic reviews with meta-analysis^77^,^84^ and one was a meta-analysis.^85^ Thirty-five reviews reported on ethnicity and/or race in trials, 13 focused on recruitment/enrolment of participants into trials, nine focused on both recruitment and reporting, eight focused on reporting and analysis of ethnic/racial variables and three on recruitment and retention.

The primary trials covered by 62 included reviews were concentrated in North America (12,317), Europe (2,694), Asia (838) and Oceania/Australia (205). Only five studies included sites in Africa. The remainder of the primary trials (33%) were from multiple sites in different countries. In terms of country representation, the majority of the trials included sites in the USA (7419). The reviews reported results from 14 427 included primary studies. The majority of the reviews (94%; n=58) had English language restrictions in the literature search.

### Quality assessment of included reviews

**Figure 2:**
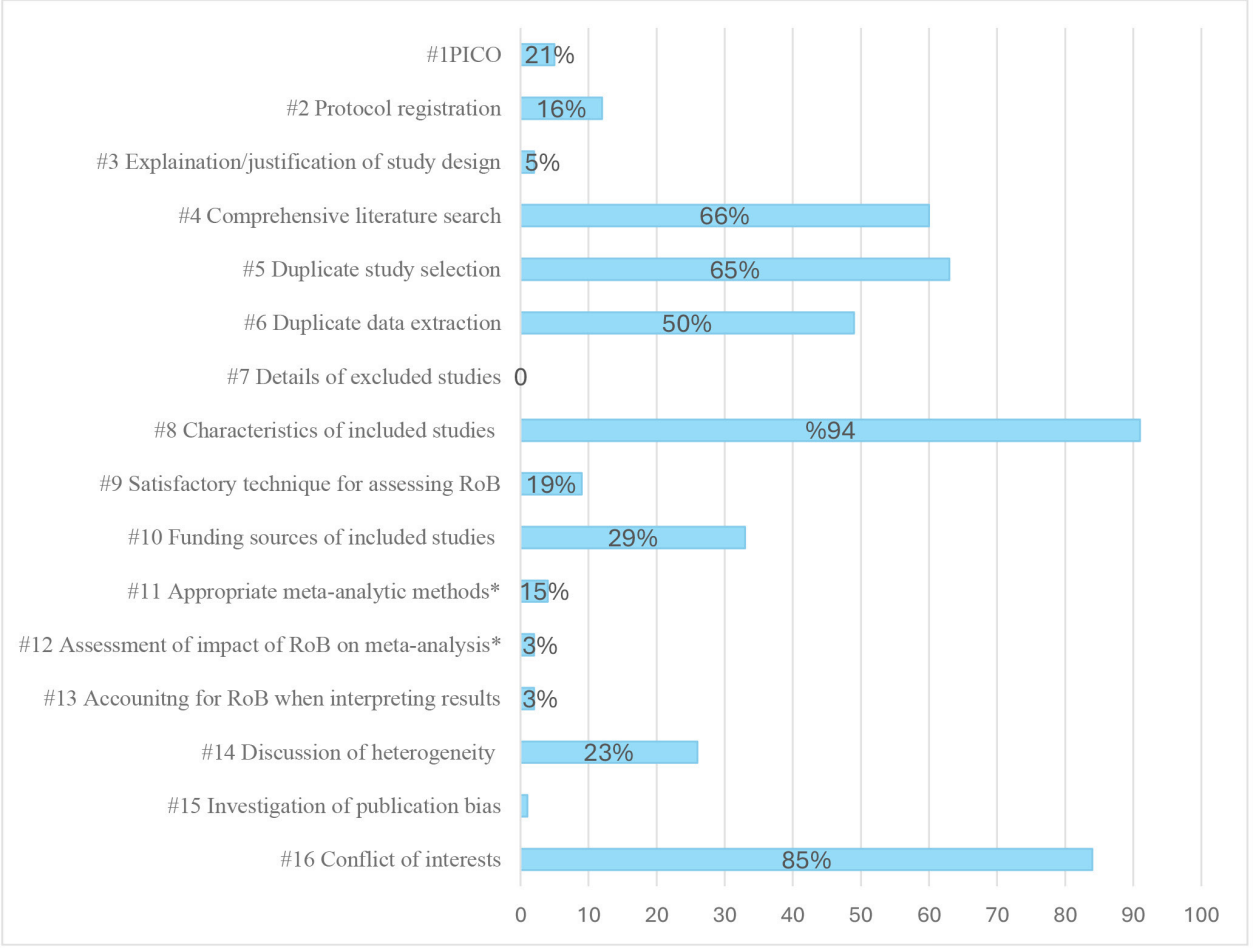
Number of reviews in percent that adhered to the items. *AMSTAR, a measurement tool to assess systematic reviews; PICO, population, intervention, comparator group, outcomes; RoB, risk of bias.

The number of reviews that adhered to each of the AMSTAR-2 items is shown in figure 2. The confidence ratings were moderate for 5 of 62 reviews (8%) and critically low for 57 (95%). None of the reviews was classified as high confidence. The best adherence was found for providing detailed information on the characteristics of included studies (item 8), declaration of conflict of interests (item 16), duplicate study selection (item 5), conducting a comprehensive literature search (item 4) and discussion of heterogeneity (item 14). The items that most reviews failed to meet were using the components of population, intervention, comparator group and outcomes when describing the research question and inclusion criteria (item 1), providing reference details of excluded studies (item 7), providing a justification for excluding individual studies (item 7), providing a justification for choice of study designs (item 3), satisfactory technique for assessing risk of bias (RoB) (item 9) and reporting the sources of funding for included studies (item 10). Only eight reviews had a meta-analysis component, so this was not assessed as an issue. For the critical domains, only 10 reviews (16%) referred to a review protocol (item 2), and 41 reviews (66%) conducted a comprehensive literature search (item 4). Only 12 reviews (19%) used a satisfactory technique for assessing the risk of bias in individual studies (item 9). However, this is not considered an issue as the reviews did not focus on the effectiveness of interventions.

### What variables related to ethnic diversity are collected and reported?

All included reviews reported on race and ethnicity as the common variables related to ethnic and cultural diversity (n=61, 98%), with the exception of one review, which focused on Indigenous peoples.^25^ Items we coded as ‘race and ethnicity’ included those that used either or both of these terms, combinations of them such as ‘ethno-racial group’,^86^ and studies that focused on broad categories such as Hispanics, Asians or Americans (African-Americans or Latino Americans).

Orkin *et al*^26^ conducted a systematic review of reporting of sociodemographic variables in trials covering 2014–2020 in high-impact journals and found that race/ethnicity was reported in 115 of 237 (48.5%) trials compared with sex or gender that was reported in 234 of the 237 trials (98.7%). In this review, other variables such as language appeared in less than 5% of the included trials. Acton *et al*^77^ examined racial and ethnic representation in acute ischaemic stroke trials published between 2010 and 2020 and found that of 12 RCTs that met the eligibility criteria (total: 7955 participants), race and ethnicity were documented in just 42% (5 publications) and only 33% (4 publications) provided a level of granularity beyond ‘White/non-White’ for race. Paul^27^ examined the rates at which RCTs published in 10 orthopaedic journals between 2015 and 2019 reported and analysed sociodemographic variables. Of 482 included trials, only 7.3% reported race and 3.1% reported ethnicity. Within this same review, articles were further subdivided into subspecialty categories. Of the 12 articles focusing on hand surgery, 8.3% reported race and no studies reported ethnicity. Canevelli’s^28^ review focused on race reporting and disparities in clinical trials on Alzheimer’s disease and found 78.4% of participants recruited in the 49 RCTs were White, 13.0% were Asian, while only a minority was constituted of Black and Hispanic participants (4.4% cumulatively). Similarly, in a systematic review of racial and ethnic diversity in orthopaedic clinical trials from 2000 to 2020, it was found that the overall distribution of 37 798 participants included in the trials reporting race or ethnicity was 81.5% White, 6.6% Black, 1.3% Hispanic, 1.5% Asian and 9.2% other.^29^ Kotlier *et al*’s^30^ review, which sought to determine the rate of reporting for sociodemographic variables in trials investigating femoral acetabular impingement and hip arthroscopy, found that of the 48 included trials, only 7 (14.6%) reported the race of study participants and less than 10% reported ethnicity. A systematic review on representation and reporting of diverse groups in trials of pharmacological agents in inflammatory bowel disease by Pathiyil *et al*^31^ found that the race of participants was only reported in 179 (28·5%) of 627 included RCTs. Further, of the 51 149 participants from these 179 trials, 43 650 (85·3%) were White, 3992 (7·8%) were Asian, 1035 (2·0%) were Black and 95 (0·2%) were Pacific Islander, suggesting under representation of ethnically diverse groups. In this same review, the authors found that ethnicity was reported in only 67 (11%) of 627 RCTs.

Patki *et al*^32^ found poor reporting of race and ethnicity in prostate cancer trials, where patients of White race were the majority of the recruited population (82.6%) compared with other groups (Black 9.8% and Asian 5.7%). While Patki *et al*^32^ observed that commercial studies were associated with improved equality, diversity and inclusion reporting, Xiao *et al*^78^ found that for COVID-19 trials in the USA, industry-sponsored trials enrolled fewer participants from ethnically diverse backgrounds compared with federally sponsored trials.

A key conclusion across the reviews included in this overview relates to limited reporting on ethnicity/race of participants. Only one review^33^ focusing on couple and family therapy intervention studies in the USA found that included trials were broadly reflective of the people who have couple and family therapy. In this review,^33^ race/ethnicity of study participants was reported in 89% of studies (175 of 196) of which about 44% (87) of the studies were classified as biased toward White, while 45% (88) were classified as inclusive. Family intervention studies included a larger proportion of racial/ethnically diverse groups compared with couple intervention studies. In family therapy studies, 55% (76 of 137) were considered inclusive, whereas only 20% (12 of 59) of couple therapy studies were considered inclusive. Of the family therapy studies containing less than 61% White samples, the majority of studies focused on just two racial/ethnic groups: African-American and Hispanic/Latino populations.

The review by Reccionni *et al*^79^ showed an improvement when it comes to reporting race/ethnicity in RCTs that involve children. The review found that data on race/ethnicity were reported for 68% (n=158 of 232) of the included RCTs in children/adolescents. Reporting of race/ethnicity also seems to have improved in COVID-19 trials. A review by Kou *et al*^34^ on reporting of health equity considerations in vaccine trials for COVID-19 found that of the 144 included trials, 64% reported on race/ethnicity. Similarly, in a systematic review and meta-analysis of racial and ethnic diversity representation in COVID-19 prevention and treatment trials in the USA, it was found that race and ethnicity were reported in 77.9% and 71.3% of the trials, respectively.^78^ The authors, however, noted that Black participants were underrepresented in COVID-19 prevention trials, though not treatment trials. Further, Hispanic or Latino participants were overrepresented in COVID-19 trials, possibly due to selection bias. Again, Talaski *et al*’s^35^ review concluded that until the current decade (2020–2030), race had not been reported for Achilles tendon pathology RCTs (8 out 68 included trials).

Beyond poor efforts to report race and ethnicity, these variables are also often reported incorrectly. For instance, a systematic review and meta-analysis by Alvarez *et al*,^80^ which investigated trends in race and ethnicity enrollment and reporting in US plastic and reconstructive surgery found that 73% of the studies did not report race or ethnicity, and among those that reported on these variables, 19.44% and 25% of those studies did so incorrectly, respectively.

As shown in online supplement additional file 2, examining the compositions of races/ethnicities in reviews more specifically, 74% (n=46) of the reviews reported that White participants were in the included primary trials, while 68% (n=42) of the reviews reported that African-American/Black were included in the primary trials. Hispanic (n=32, 52%) and Asian (n=35, 56%) were the other major ethnic groups represented in trials.

A review focusing on transparency of racial participation reporting in RCTs of minimally invasive surgical techniques included 496 trials, of which racial information was reported in 20 (4.03%).^36^ Of the 20 trials reporting racial information, White participants were in the majority (84.5%) followed by African American/Black (7.9%), Latin/Hispanic (2.1%) and Asian (1.2%). The review concluded that among trials assessing minimally invasive surgical techniques over the past 30 years, data on ethnicity are poorly reported. Tahhan’s^37^ review examined enrolment of older patients, women and racial/ethnically diverse groups in contemporary acute coronary syndrome clinical trials. Of the 460 included trials, the distribution of ethnic groups was reported in only 99 trials (21.5%). In trials with reported data, 15.0% of the trial participants were non-White, which increased from 12.0% in 2001–2006 to 14.0% in 2013–2018. Black patients represented 3.7% of all patients during the entire trial time frame, Asian patients represented 9.6%, and Hispanic patients represented 7.8%. Trends in the representation of Black patients remained unchanged from 2001 to 2006 (5.2%) to 2013–2018 (4.9%), while the enrolment of Asian and Hispanic patients increased from 2001 to 2006 to 2013–2018 (from 1.9% to 10.8% for Asian patients and from 5.4% to 14.5% for Hispanic patients). Mendoza *et al*^38^ conducted a review on minority inclusion in trials of panic disorder including 45 trials of which 21 trials with 2687 participants reported ethnic and racial data for their sample. Of the 21 studies reporting information on ethnicity and race, 82.7% were European American/non-Hispanic White, 4.9% were African American/Black, 3.4% were Hispanic, 1.1% were Asian American and 1.4% were another ethnicity. The remaining 6.5% was simply classified as other/non-White.

### What methods are used to recruit and retain participants from ethnically diverse backgrounds?

Online supplement additional file 2 provides details on the recruitment, retention and analysis of variables relevant to ethnicity.

### Recruitment

Fourteen reviews (23%) reported on strategies to recruit and support ethnically diverse participation in trials (see figure 2). Six reviews^39^,^44^ reported on community engagement with ethnically diverse populations as the predominant recruitment strategy used by trialists. Five reviews reported advertisement in mainstream media and social media^38 40 41 45 46^ and four reviews each reported face-to-face recruitment^39 41^ and cultural targeting as the predominant recruitment strategies used by trialists. Community engagement recruitment strategies included the use of bilingual and bicultural workers and employing bilingual research assistants.^44^ Cultural targeting strategies included using interpreters and translating trial materials into the participants’ preferred languages and providing the option for interventions to be delivered in participants’ preferred language.^44^

Carroll *et al*^40^ examined recruitment and retention strategies for underserved populations in physical activity interventions in primary care and community settings and found that successful recruitment strategies consisted of partnering with respected community stakeholders and organisations, use of ethnically and linguistically trained trial staff and use of multiple advertising channels. A review of strategies to recruit ethnically diverse participants found that both proactive (face-to-face) and reactive recruitment strategies (eg, collaboration with community leaders, printed material and broadcast media) and providing incentives, building rapport and trust and employing ethnically and culturally diverse research staff were helpful to recruitment.^41^ Heller^43^ reported that community engagement strategies including engaging service providers, hospitals and/or participants in the underserved communities are a major strategy to overcome barriers that diminish the opportunity for underrepresented participants to enrol in clinical trials. Haughton^42^ also reported that community engagement, community-based participatory research and research partnerships show promise for increasing inclusion of diverse ethnic groups in trials. Similarly, Long *et al*^81^ observed that the inclusion and adoption of non-traditional recruitment strategies can boost the inclusion of diverse and under-represented groups in trials.

Other recruitment strategies included clinical referral,^38 41 46^ community presentations,^41 45^ peer-to-peer sampling,^42^ use of technology,^25 42 47^ provision of incentives^39 41^ and research partnership.^42 48^ Regarding the use of technology for recruitment, Rosenbaum^47^ conducted a review of racial and ethnic minority enrolment in trials of behavioural weight loss using technology and found a significantly higher racial minority enrolment in studies that used smartphones in their interventions compared with those that did not. The review further found that across technology types, enrolment did not differ based on whether in-person visits were required (t (51) =−0.55, p=0.585). Similarly, Haughton^42^ reported that the use of technology to both recruit and deliver behavioural weight loss interventions shows promise for increasing inclusion of diverse samples.

Nicholson^39^ examined recruitment and retention strategies in trials with low-income and ethnically diverse groups covering the periods 2004–2014 and found that both proactive and reactive strategies are helpful in recruiting persons of different ethnic or cultural backgrounds. The proactive strategy that was reported in this review comprised the use of face-to-face interactions. Collaboration with key community leaders was reported as an effective reactive strategy. The review concluded that ethnically diverse persons are more inclined to participate in research when community leaders support the research being conducted and are actively disseminating information about the trial to their community.

In a systematic review of barriers and facilitators to participation in RCTs by Indigenous people from New Zealand, Australia, Canada and the USA, Glover^25^ found that common factors that were seen as facilitators included partnership and relationship building, culturally appropriate trial design, employing Indigenous staff, targeted recruitment techniques and appropriate trial materials. English language as an eligibility criterion was identified as a barrier to inclusion of participants from diverse ethnic backgrounds.^48^

On the whole, the findings reveal that the effectiveness of these strategies are rarely evaluated.^39^ Only two reviews^38 42^ provided information on effectiveness of recruitment strategies. One review assessed the effectiveness of smartphone as a recruitment strategy compared with other strategies and found that weight loss trials that incorporated smartphone use had greater enrolment of ethnically diverse participants than trials that did not incorporate smartphones.^42^ Mendoza *et al*^42^ analysed two recruitment strategies, clinical referral and advertising, but neither of these methods were correlated with improved participation. The findings from one review showed that the use of technology (smartphones) for recruitment increased the enrolment of diverse groups.^47^

### Retention

Retention strategies highlighted by the reviews include frequent follow-ups on participants to check how they are doing in trials,^25 39 40^ designing and implementing trials at the convenience of participants, provision of incentives,^25 40 41 44 45^ use of community-based techniques^44^ and use of tailored approaches and culturally appropriate interventions.^25 40 44^

Nicholson^39^ reported that personal and continuing follow-up on participants is important in retention. Glover *et al*^25^ found that including Indigenous groups from the outset in the early design phase of the trial, with Indigenous groups guiding research, and research driven by needs identified by the community were all key facilitators of retaining participants in trials. One of the main themes highlighted by the included reviews as a barrier to retention is the lack of tailored approaches from researchers.^44^ To recruit and retain participants from ethnically diverse backgrounds in trials, consideration should be given to the communities’ cultural requirements in the recruitment process, and consideration of various strategies including cultural, linguistic needs and researcher’s awareness of the cultural expectations.^44^

Teague *et al*’s^87^ systematic review and meta-analysis of retention strategies found that studies employing more emerging retention strategies such as social media and SMS to assist in tracing participants lost to follow-up and the use of trial websites and social media profiles for keeping participants up-to-date with trial news and events were associated with improved retention rates. In the case of the use of incentives, reviews focusing on retention have found that incentives are associated with an increase in overall retention rates.^88^

Carroll *et al*^40^ found that successful retention strategies included efficient administrative tracking of participants, persistence, skilful teamwork, demonstrating a positive, caring attitude towards participants and having bicultural, bilingual and/or ethnically matched trial team members. Cui^45^ conducted a review of recruitment and retention in obesity prevention and treatment trials targeting ethnically diverse and low-income children. The review found four key strategies for retention of trial participants: intervention design (ie, designing culturally appropriate intervention activities and setting developmentally appropriate goals for participants); provision of incentives (including grocery gift cards, gifts, cash, food, recipe books and exercise equipment and regular communication with participants, such as thank-you notes, postcards or project newsletters); participant convenience (ie, providing transportation support to and from intervention activities or data collection, make-up sessions for missed intervention sessions, upcoming event reminders, childcare services and optional days or home visits for data collection); and participant tracking (eg, home visits for data collection).

A key conclusion emerging from the included reviews was that successful recruitment and retention are grounded in having a well-trained, bicultural and/or ethnically and linguistically matched research team, cultural tailoring of the intervention, strong community partnerships/engagement and strong interpersonal skills.^39 42 43^ Some reviews raised a concern that retention strategies are under-reported, less detailed when reported and their effectiveness rarely rigorously tested.^39^ Recommendations from the reviews to aid recruitment and retention of participants in trials included the need to include trial sites in geographic regions that are accessible to participants from diverse ethnic backgrounds; the need to reduce obstacles to inclusion, such as providing trial information in appropriate languages and using outcome measures that are translated into, and validated in, other languages^48^; and a need for training and support provisions for the trialists to improve and build up their recruitment skills to facilitate enhanced ethnic recruitment accruals.^44^ Glover *et al*^25^ concluded that in order to increase recruitment and retention, trials of interventions with Indigenous populations must be culturally tailored and use frameworks grounded in Indigenous worldviews.

### What types of analyses are conducted on ethnicity variables?

Of 18 review articles that focused on analysis of ethnic/race variables, six^928 49^,^52^ reported that there was no prior analysis plan for ethnicity. The findings indicate that sub-group analysis by ethnicity is seldom done in trials, and following from that, the method of analysis of ethnicity/race in trials is rarely described in publications of trials.^42 53^ Riaz *et al*^85^conducted a meta-analysis of 286 trials including 9552 older adults with prostate cancer and found that while racial and ethnic subgroups were reported in 69.2% and 26.2% of trials, respectively, analysis of clinical outcomes by race was reported in 3.1% of trials among those with reporting with no analysis done for ethnicity.

An improved understanding of the differences between subgroups (race/ethnicity) is important for improving the risk/benefit profile for a wide range of therapeutic efforts. Haughton^42^ found that of 60 studies that included more than one racial/ethnic group, only eight included sub-group analyses of weight loss outcomes by race/ethnicity and two included sub-group analyses of predictors of intervention attendance by race/ethnicity. Hirano^53^ conducted a systematic review on reporting, representation and subgroup analysis of race and ethnicity in published clinical trials of atopic dermatitis and found a priori subgroup analyses by ethnicity was lacking across the 78 included trials. The review concluded that there is a need for greater representation of participants from diverse ethnic backgrounds, better reporting and a priori subgroup analyses in trials of atopic dermatitis. Schick^50^ conducted a systematic review of inclusion in trials of pharmacological treatments for alcohol use disorder and found that of 102 trials, only 5.9% of articles conducted sub-group analyses to examine differences in treatment outcomes by sex or race/ethnicity.

In a review of inclusion of participants from diverse ethnic backgrounds and women in cancer clinical trials, Kwiatkowski *et al*^49^ reported that of 277 included trials, only 11.1% (n=31) specified a subgroup analysis across ethnicity. The authors further found that statistical precision of results for subgroups depends on the numbers in those groups, meaning oversampling of a particular ethnic group may be necessary to ensure results are ‘generalisable’ for the intended treatment groups. Paul’s^27^ review which included 482 trials found that only six studies did a subgroup analysis by race/ethnicity. Of the six studies, two showed differences based on race/ethnicity. One trial found through a logistic regression multivariable analysis that White patients were more likely to respond to patient-reported outcome surveys compared with Black patients (OR of 2.03) after orthopaedic surgery. Griffin^54^ found that while race and ethnicity were each reported by 6.2% and 3.8%, respectively, of the 209 included trials, there was little analysis of race or ethnicity.

## Discussion

The findings point to limited ethnic diversity in trials. A key conclusion across the reviews was that trial participants are not representative of the ethnic diversity of the target or general population, with only modest improvement over time. While some reviews found some level of improvement in trial diversity,^33^ in all reviews, ‘White’ was the most common ethnicity reported. This aligns with other studies that have shown that White individuals are more often recruited and retained in trials compared with other ethnic groups.^26^ The findings further show that most trials under-recruit participants from diverse ethnic backgrounds, which perpetuates health inequalities. This suggests that guidelines for reporting on the inclusion of ethnic diversity in trials have yet to achieve their intended goals.

Recruitment strategies commonly reported by the reviews were community engagement, advertisement, face-to-face recruitment, cultural targeting, clinical referral, community presentation, use of technology, incentives and research partnership with communities. Strategies like cultural targeting, community engagement and incentives were found to support all trial participants. Further, barrier reduction strategies such as the use of researchers or clinical staff who spoke the participants’ preferred language or translation of trial materials were key ways in which trialists achieved participation and retention of diverse groups in trials.^39 41^ This corroborates the findings from a systematic review and meta-analysis,^87^ which analysed 95 retention strategies in cohort studies and found that studies using barrier-reduction strategies (eg, offering childcare services, addressing language barriers, adapting materials for different languages) retained 10% more of their sample (95% CI (0.13 to 1.08); p = 0.01) compared with studies using follow-up/reminder strategies (95% CI (− 1.19 to − 0.21); p = 0.02).

Reviews often reported that language proficiency as an eligibility criterion is a barrier to inclusion of diverse groups in trials.^39 44 48^ This aligns with a review by Isaac *et al*’s^55^ on optimising the recruitment of participants from diverse ethnic backgrounds to telehealth diabetes trials, which found that a key characteristic of high diversity-recruiting studies was an emphasis on languages other than English. Cultural mistrust/distrust, discrimination and researcher’s inability to understand certain values or expressions underserved populations may hold were all identified as barriers to participation in trials.^38^ One way to address the issue of trust is by collaborating with ethnic community organisations, which has been found to help participants to overcome the stigma and mistrust associated with the research process.^44^ Williams *et al*^89^ conducted a trial to determine how to improve recruitment of African Americans into anxiety disorders research and found that assurances of confidentiality, adequate compensation and appreciation of the African-American community and their culture contributed to increasing African-American participation in the trial.

In terms of gaps in the literature on recruitment and retention, some reviews noted that researchers often employed limited strategies to enhance the recruitment level.^44^ Other reviews also highlighted that the full extent of the use of strategies was not described well in the publications. These findings indicate that there is a need for wider training and support for trialists to enhance and build up recruitment skills to facilitate the recruitment of ethnically diverse populations into trials.^44^

The findings point to a limited focus on the analysis of variables relevant to ethnicity in trials even when they are reported. The 16 reviews that provided information on analyses of variables relevant to ethnicity concluded that, analysis, and in particular, a prior analysis plan was lacking in most trials.^49 50 52 54^ This suggests that despite increased awareness of the importance of ethnicity on patient outcomes, there has been little increase in the frequency at which they are reported and analysed in trials. This means that trialists should initiate discussions about ethnic diversity reporting, including analysis, in the early stages of trial development as well as justifying a priori the goals for including ethnic diversity. As noted by Cwalina,^29^ the inclusion of ethnicity data in trials should be based on specific sub-group analysis and necessary statistical power. Kong *et al*^56^ note that given the trials provide information about safety and efficacy, extending the findings from trials to different ethnic populations is imperative for providing clinical care. In short, trialists need to pay more attention to ethnic diversity when designing and reporting their studies and should plan their analyses accordingly to better enumerate how these factors may influence clinical outcomes.^52^

### Strengths and limitations

This synthesis followed a preregistered protocol and was conducted in accordance with established criteria for umbrella reviews.^90^ Further, in this review, we employed rigorous methods to search and synthesise evidence from existing reviews. Study selection, data extraction and quality assessment were all conducted by two independent reviewers, thus increasing the internal validity of the review findings. Similarly, the Stakeholder Advisory Committee, which included representatives from four ethnically diverse consumers, also provided expert advice on the reviews processes, thus contributing to the internal validity of the review.^91 92^

We acknowledge that the primary studies that formed the unit of analyses of the included reviews are overwhelmingly from the USA, which is a limitation of primary research rather than our review per se. Another major limitation of the primary studies in the included reviews was the poor quality of the reporting of retention and recruitment strategies and their effectiveness, which limits our ability to comment on the effectiveness of the strategies employed by trials to include participants from ethnically diverse backgrounds in research. Further, terminology, best practice and cultural norms change rapidly in this field. Therefore, the review draws on content that is 23 years old—which may be stale—and is likely to change rapidly still. Finally, we limited our search to review papers published in English and might have missed reviews published in other languages.

## Conclusions

This overview of reviews provides a summary of the current evidence and gaps in evidence related to ethnicity reporting and the inclusion of ethnic diversity in trials. It also provides the opportunity to reflect on what needs to be considered by trialists in relation to recruitment and retention strategies in trials and analysis of variables related to ethnicity to consider in trials. A major gap in the review literature was the lack of reviews exploring the effectiveness of recruitment strategies in increasing enrolment of participants from ethnically diverse backgrounds in trials. Overall, the findings show that ethnicity and the inclusion of ethnic diversity are poorly reported across trials. When reported, the relative proportions of diverse groups (eg, Black, Asian, Hispanic/Latino participants) are under-represented with White participants making up between 70% and 95% of most samples.

To conclude, this umbrella review has shown that underreporting, recruitment and poor analysis of ethnicity in clinical trials is persistent and poses a significant threat to health equity. In line with the findings, box 1 summarises the key messages and recommendations for improving the inclusion of ethnic diversity in trials.

Box 1 **Key messages and recommendations for recruitment, reporting retention and analysis of ethnicity in trials.**

***Collecting and reporting variables relevant to ethnicity in trials***

- There is the need for more detailed reporting of ethnicity within trials.
- Study participants should be able to define their preferred ethnic identity.
- Widespread and consistent reporting of ethnic diversity and ethnicity assignment measures are needed.
- English language or majority language should not be used as an inclusion criterion as this limits inclusion of groups.
- Ethnicity variables should be used to explore social issues and experiences, not biological facts.
- When reporting ethnicity data, include descriptors such as ‘self-reported data’ to clarify the source of the information.
- Study sponsors and journal editors can play an important role in improving the quality of reporting of ethnicity.
- There is the need to develop guideline on the usage of race and ethnicity reporting terms in trials.

***Methods for recruitment and retention of participants from ethnically diverse backgrounds in trials***

- Successful recruitment and retention strategies are grounded in having a well-trained, bicultural/multicultural and/or ethnically and linguistically matched research team, cultural tailoring of the intervention, strong community partnerships and interpersonal skills.
- Recruitment of participants from ethnically diverse backgrounds involves consideration of communities’ cultural requirements in the recruitment process; trialists should consider the cultural and linguistic needs and researcher’s awareness of the cultural expectations.
- There is a need for development and validation of a tool to provide trial recruiters with a simple, practical means of assessing language proficiency for trial participation to help minimise the possibility of patients being unfairly excluded based on arbitrary judgements.
- Study designers should consider reducing obstacles to inclusion such as providing study information in appropriate languages and using outcome measures that are translated into, and validated in, other languages.
- The development of guidelines for reporting recruitment and retention would be a first step in improving the quality of information reported in this area.
- Trialists would benefit from a culturally sensitive recruitment training programme to enhance recruitment and retention of participants from ethnically diverse backgrounds in trials.
- Recruitment by ethnicity should be included in quality assessment frameworks for trials and must be considered a measure of study quality.

***Analysis of ethnicity variables***

- The review clearly demonstrates a need for better reporting and careful methodological work to conduct appropriate subgroup analysis of ethnicity in trials where it makes sense.
- Statistical analyses should align with the study’s conceptualisation and operationalisation of ethnicity.
- The decision to include ethnicity data in analyses should be based on specific hypotheses and statistical power analysis.
- There is the need to clarify the purpose and justification for including ethnicity variables in analyses *a priori*.

## supplementary material

10.1136/bmjopen-2024-084889 online supplemental file 1

10.1136/bmjopen-2024-084889 online supplemental file 2

## Data Availability

All data relevant to the study are included in the article or uploaded as supplementary information.
