# Supplementary material for: Recruitment, retention and reporting of variables related to ethnic diversity in randomised controlled trials: an umbrella review
Source: BMJ Open. 2024 Aug 9;14(8):e084889. doi: 10.1136/bmjopen-2024-084889 (PMC11340254; doi:10.1136/bmjopen-2024-084889)
Supplement: online supplemental file 1 [file bmjopen-14-8-s001.pdf]

Additional file 1: Characteristics of reviews

Author	Review design	Focus area of review	Populations	No. of included studies	No. of participants	Search period covered	Countries where trials were conducted (n*)
Charrow (2017) [9]	Systematic review	Reporting	Dermatologic conditions	626	Not specified	2010-2015	US (164); non-US (462)
Glover (2015) [25]	Systematic review	Recruitment	Indigenous people	53	Not provided	Papers published prior to March 2012	USA (35) NZ (8) Australia (6) Canada (4)
Orkin (2021) [26]	Systematic review	Reporting	General	237	Not provided	2014-2020	Not specified
Acton (2022) [77]	Systematic review and meta-analysis	Reporting	Acute ischemic stroke	12	7,955	2010-2020	US and Canada
Paul (2021) [27]	Systematic review	Reporting and analysis	Orthopaedics	482	Not specified	2015 - 2019	US (482)
Canevelli (2019) [28]	Systematic review	Reporting and analysis	Alzheimer's disease	49	21,000	1996-2018	Multi-sites
Cwalina (2022) [29]	Systematic review	Reporting	Orthopaedic	1,043	184,643	2000-2020	United States (361), North America (88), Europe (388), Asia (136), Australia or New Zealand (53), Africa (4), Middle East (8)
Kotlier (2024) [30]	Systematic review	Reporting	Femoral acetabular impingement	48	Not provided	2011-2023	Not provided
Pathiyil (2023) [31]	Systematic review	Reporting	Inflammatory bowel disease	627	108986	1995-2023	Cross-continental (189) Europe (431) North America (239)

							Asia (207)<br>Oceania (73)<br>Africa (56)<br>South America (35)
Patki (2023) [32]	Systematic review	Reporting	Prostate cancer	54	19 039	2010-2020	USA (36)<br>Multinational (43)<br>UK (8)<br>Canada (8)<br>Netherlands (6)<br>Japan (5)<br>France (4)<br>Italy (4)
Xiao (2024) [78]	Systematic review and Meta-analysis	Reporting	COVID-19 trials	122	176 654	2019-2022	US (122)
Tseng (2021) [33]	Systematic Review	Recruitment	Couple and family therapy intervention	196	Not provided	2014-2019	USA (196)
Riccioni (2023) [79]	Systematic review and meta-analysis	Reporting	Attention-deficit/hyperactivity disorder (ADHD) medications	310	44,447	2014-20223	US (215)<br>Europe (37)<br>Asia (21)<br>Other (24)
Kou (2024) [34]	Systematic review	Reporting	COVID-19 vaccine trials	144	88,772,	2020-2023	Americas (27)<br>Europe (31)<br>South-East Asia (17)<br>Western Pacific (43)<br>Africa (3)<br>Multiple (18)
Talaski (2024) [35]	Systematic review	Reporting	Achilles tendon pathology	68	Not provided	1999-2023	US (6)<br>Outside US (61)<br>Unknown (1)
Alvarez (2024) [80]	Systematic review and meta-analysis	Reporting	Plastic and reconstructive surgery	36	7281	2012-2022	US (36)

Sayyid (2021) [36]	Systematic review	Reporting	Minimally invasive surgical techniques	496	Not specified for full sample; 17 trials with racial data (12,543)	Up to December 2017	USA, UK, Canada, Brazil, Australia.
Tahhan (2020) [37]	Systematic review	Recruitment	Acute coronary syndrome	460	1,067,520	2001-2018	USA<br>Western Europe<br>Rest of the world<br>Multiregional
Mendoza (2012) [38]	Systematic review	Recruitment; Reporting	Panic disorder	47	2,687	1993-2010	US and the Canada (18), US (1)
Nicholson (2015) [39]	Systematic review	Recruitment and retention	Low-income and minority populations	165	Not provided	2004-2014	USA (165)
Carroll (2011) [40]	Systematic review	Recruitment and retention	Physical activity interventions in primary care and community settings	38	8,790	1998-2010	US (38)
Ibrahim (2013) [41]	Systematic review	Recruitment	Minority persons	26	Not provided	1995-2012	USA (23)<br>Australia (2)<br>Canada (1)
Haughton (2018) [42]	systematic review	Recruitment; Reporting	Lifestyle weight loss intervention interventions	94	29,320	2009-2015	USA (94)
Heller (2014) [43]	Systematic review	Recruitment	General	21	Not provided	2001-2013	USA (21)
Masood (2019) [44]	Systematic review	Recruitment	Physical illness (diabetes and ischemic heart disease) and mental disorders	21	8,687	Up to 2018	UK (21)

Cui (2015) [45]	Systematic review	Recruitment and retention	Obesity prevention and treatment - children	43	Not provided	Not provided	not provided
Burnette (2022) [46]	Systematic review	Recruitment reporting	Eating disorder psychotherapy treatment	58	4,864	1985-2020	USA (58)
Long (2024) [81]	Systematic review and meta-analysis	Recruitment, reporting and analysis	peripheral artery disease	59	8302	2012-2022	Germany (12)<br>US (11)<br>China (7)<br>Other (24)
Rosenbaum [47]	Systematic review	Recruitment	Behavioural weight loss (BWL) treatment,	71	Not provided	Not specified	US
Somerson (2014) [48]	Systematic review	Reporting	Orthopaedic	158	37,625	2008-2011	USA (158)
Kwiatkowski (2013) [49]	Systematic review	Reporting	Cancer	277	329,671	2001 - 2010	US (277)
Schick (2020) [50]	Systematic review	Reporting and analysis	Pharmacological treatments	102	Not provided	1994-2019	USA (54)
Chen (2022) [51]	Systematic review	Reporting	Dermatologic conditions	392	Not provided	2015-2020	US (100); non-US (292)
Begic (2019) [52]	Systematic review	Reporting and analysis	Anesthesiology trials	732	Not specified	2014-2017	USA (169); Canada (52); Denmark (49); UK (38); Korea (37); China (37); Germany (36); France (36); Australia (25); Netherlands (25); Other (228)
Hirano (2021) [53]	Systematic review	Reporting and analysis	Atopic dermatitis	78	Average number of participants 203	2000-2009	US (78)
Riaz (2023) [85]	Meta-analysis	Reporting and analysis	Prostate cancer	286	104 205	1989- 2020	US (65)<br>Outside US (115)

							International with US participants (62)<br>Region unspecified (44)
Griffin (2021) [54]	Systematic review	Reporting	Not specified	209	Not specified	2015-2019	USA (77)<br>China (25)<br>Other (25)<br>Australia (17)<br>South Korea (15)<br>Japan (15)<br>Netherlands (8)<br>Canada (4)<br>UK (8)<br>Thailand (6)<br>Denmark (4)
Isaacs (2016) [55]	Systematic review	Reporting	Type 2 diabetes	79	12,916	2000-2015	United States (59), Australia (7), Canada (7), the United Kingdom (5), both the United States and Canada (1).
Kong (2021) [56]	Systematic review	Reporting	Epilepsy	230	39,576	1988 - 2019.	Not specified
Zhang (2013) [57]	Systematic review	Recruitment	Cardiovascular diseases	250	1,103,694	1997-2010	USA (250)
Vyas (2018) [82]	Systematic review and meta-analysis	Recruitment	Dementia	96	37,278	2000-2017	not specified
Shaw (2021) [58]	Systematic review	Recruitment; Reporting	Brain health and prevention trials	42	100,748	2004-2020	USA (42)
Wilder (2016) [59]	Systematic review	Recruitment	Hepatitis C	314	Not provided	2000-2011	North America (69)<br>Europe (143)<br>Multinational (102)
Ricardo (2022) [60]	Systematic review	Recruitment	Nail ssoriasis	45	12,943	Not specified	US (22) non-US (23)

Rencsok (2020) [61]	Systematic review	Recruitment; Reporting	Prostate cancer	72	893,378	1987-2016	US (72)
Polo (2019) [62]	Systematic review	Recruitment; Reporting and analysis	Depression	342	61,283	1981-2016	USA (342)
Onuorah (2022) [63]	Systematic review	Recruitment; Reporting	Multiple sclerosis	44	42 to 2,244	1995-2020	Brazil, Ireland, South Africa, the United Kingdom, United States.
Nalven (2021) [64]	Systematic review	Recruitment; Reporting	Opioid pharmacological treatment trials:	50	9,124	Up to March 2020	USA (50)
Minocher Homji (2011) [65]	Systematic review		Cardiovascular disease	45	140,764	1980-2009	USA (5) UK (3) Canada (2 Australia (1)
Franzen (2021) [66]	Systematic review	Reporting	Alzheimer's disease	101	Not provided	2001-2019	North America (80), Europe (61%), Asia (37), Oceania (33); South America (15), Africa (7).
Fletcher (2022) [67]	Systematic review	Reporting	BCG-unresponsive non-muscle invasive bladder cancer	27	1.673	1998-2021	US (21) Canada (6)
Falasinnu (2018) [68]	Systematic review	Reporting	Systemic lupus erythematosus	193	Not provided	1997-2017	US (79) Others (114)
Eichel (2021) [69]	Systematic review	Reporting	Mindfulness research	94	3,582 (42/94 RCTs)	Not specified	US (45) Canada (8) Europe (31) UK (7) Iran (6) Israel (1) China (2) Australia (1)

Buffenstein (2023) [83]	Systematic review and meta-analysis	Reporting	Different kinds of diseases	2977	607,181	2008-2019	US (2977)
Chopera [70]	Systematic review	Reporting	Chronic obstructive pulmonary disease (COPD)	36	2996	from inception to 31 December 2022,	US (14)<br>UK (8)<br>Australia (9)<br>Canada (5)
De Jesús-Romero (2024) [71]	Systematic review	Reporting	cognitive behavioral therapy (iCBT)	62	17,210	1966-2022	US (17); Germany (12), Australia (10), Sweden (10), Netherlands (6), China (2), Finland (2), UK (2), Canada (1), Colombia (1), Ireland (1), New Zealand (1), Switzerland (1)
Delma (2023) [72]	Systematic review	Reporting	upper-extremity	481	Not provided	2000-2021	North America (158)<br>Europe (184)<br>Asia (91)<br>Middle East (17)<br>South America (10)<br>Australia (12)<br>Africa (6)<br>Oceania (3)
Gala (2023) [73]	Systematic review	Reporting	Obesity	11	2129	From inception to 2022	US (11)
Maria Guzmán (2023) [74]	Systematic review	Reporting	self-injurious thoughts and behaviors (SITBs)	525	Not provided	U.S. (n = 217) and international (n= 308)	80.8% (n = 424) of RCTs were conducted in high-income countries (e.g., United States, England, Norway, and Australia), while only 6.7% (n=35) were conducted in middle-

							income countries (e.g., India, Brazil, and China)
Issa (2023)[75]	Systematic review	Reporting and analysis	Spine surgery	278	Not provided	2012 - 2022	US (67),<br>China (30),<br>South Korea (17),<br>Japan (N=14)<br>Multiple countries (8)<br>Other (142 from 32 countries)
Pelton (2024) [84]	Systematic review and Meta-analysis	Reporting	Crohn’s disease	67	20 013	Not provided	Not provided
Steventon (2024) [76]	Systematic review	Reporting	systemic anti-cancer therapies (SACT) for gynecological cancers,	26	17,041	2012-2022	Of 5,478 research sites, 80.1% were located in North America, 13.0% in Europe, 3.4% in East Asia, 1.3% in the Middle East, 1.3% in South America and 0.8% in Australasia.
