# Supplementary material for: Recruitment, retention and reporting of variables related to ethnic diversity in randomised controlled trials: an umbrella review
Source: BMJ Open. 2024 Aug 9;14(8):e084889. doi: 10.1136/bmjopen-2024-084889 (PMC11340254; doi:10.1136/bmjopen-2024-084889)
Supplement: online supplemental file 2 [file bmjopen-14-8-s002.pdf]

Additional file 2: Characteristics of ethnicity, recruitment and retention strategies and analysis of ethnic variables

Author	Ethnicity-related variables collected	Ethnic/racial categories	Recruitment strategies	Retention strategies	Analysis of ethnicity/race
Zhang (2013) [57]	Race/ethnicity	Whites, African-Americans, Hispanics, and Asians	N/A*	N/A	N/A
Vyas (2018) [82]	race/ethnicity	Asian, African/Caribbean black, Hispanic/Latino, and other	N/A	N/A	Yes
Tseng (2021) [33]	race/ethnicity	White, African American, Hispanic/Latino/a participants, Asian/Pacific Islander/Native Hawaiian participants, American Indian/Native Alaskan participants.	N/A	N/A	No
Tahhan (2020) [37]	race and ethnicity	Black, Asian, Hispanic, Non-white	N/A	N/A	No
Somerson (2014) [48]	race and ethnicity	Hispanic, African-American	N/A	N/A	No
Shaw (2021) [58]	race and ethnicity	White, Black or African American. American Indian or Alaska Native, Hawaii Native or Pacific Islander	N/A	N/A	N/A
Schick (2020) [50]	race/ethnicity	White, Black/African American, Hispanic/Latinx, Asian, American Indian/Alaska Native, Multiracial	N/A	N/A	No
Sayyid (2021) [36]	race	White/Caucasian, Black/African American, Asian, Latin/Hispanic	N/A	N/A	Yes,

Wilder (2016)[59]	race	African American, White	N/A	N/A	No
Rosenbaum (2017) [47]	race, ethnicity	White, African-American/Black, Asian American, American Indian/Alaska Native, Hispanic (7.7%), Other racial minorities .	Technology (e.g. smartphones)	N/A	N/A
Ricardo (2022) [60]	race, ethnicity	White, Asian, black/African American, American Indian/Alaska Native, and Native Hawaiian/Other Pacific Islander, Asian, Hispanic or Latino. Multiracial	N/A	N/A	N/A
Rencsok (2020) [61]	race, ethnicity	White, Black/African American, American Indian, Alaska Native, Pacific Islander, Hispanic, Latino	N/A	N/A	No
Polo (2019) [62]	race, ethnicity,	European American, African American, Latino, Asian America, Native American, Native Hawaiians/Pacific Islanders or Native Americans/Native Alaskans, multiracial	N/A	N/A	N/A
Paul (2021) [27]	race, ethnicity	White/Caucasian, Black/African American, Asian Hispanic	N/A	N/A	Yes
Onuorah (2022) [63]	race/ethnicity	White, Black, Asian, and, in one case, Native American/Alaskan Native, Hispanic	N/A	N/A	Yes
Nicholson (2015) [39]	ethnicity	African Americans/Blacks Latinos/Latinas/Hispanics, Native Americans, Multiethnic Minorities	Face-to-face interaction, research staff training, incentives, Cultural targeting; Community engagement community-based participatory research	Incentives, personal approach, utilizing a dedicated phone line, project identity and logos, participant convenience, repeated contact with participants, volunteers from the community as community health advisors.	N/A
Nalven (2021) [64]	race and ethnicity	White, Black, Hispanic/Latinx, American Indian/Alaska Native, Asian, Native	N/A	N/A	Yes

		Hawaiian/Other Pacific Islander, multiracial			
Mendoza (2012) [38]	ethnicity, race	European American/non-Hispanic White, African American/Black, Hispanic, Asian American, and another ethnicity.	medical referral, Advertisements	N/A	No
Masood (2019) [44]	Ethnicity	Ethnic minority populations	Engagement with community and family: Working with religious leaders; Collaborating with ethnic community organisations; Self referrals and assistance from family and carers; Ethnic matching, Linguistic matching, language interpretation, Follow-up arrangements, home recruitment visits, telephone follow-up calls	The main recruitment strategies used in trials were recruitment from ethnically diverse areas; recruitment from community/religious organisation; ethnic matching at sites; interviews conducted in preferred language; telephone follow-up and interpreting service.	Yes
Kwiatkowski (2013) [49]	race/ethnicity	White, African American, Asian, Hispanic, Native American, and Other.	N/A	N/A	Not specified
Kong (2021) [56]	race, ethnicity	Race as White, Black/African American, Asian, American Indian/Alaskan, and Native Hawaiian/Pacific Islander. Ethnicity as Hispanic or non-Hispanic	N/A	N/A	No
Isaacs (2016) [55]	ethnic minority, English language	Spanish, Cantonese<br><br>Of the studies that included language	N/A	N/A	Yes

		proficiency as an eligibility criterion, there was little consistency in the way that this was defined.			
Ibrahim (2013) [41]	ethnic or cultural groups	African American, Middle Eastern, Chinese, Latino, Hispanic	Direct face to face interactions of research personnel with potential participants, presentations<br><br>Collaboration with community leaders; referrals by healthcare professionals; snowballing; printed material; and broadcast media.	N/A	No
Minocher Homji (2011) [65]	Ethnicity	Asian, White or Black/African American, Pacific Islander, First Nations status	N/A	N/A	N/A
Heller (2014) [43]	Race, ethnicity	African Americans; Hispanic/Latinos, Racial/ethnic minority women,	Engagement of providers, hospitals, and/or participants in the underserved communities.<br><br>Engagement of community members	N/A	N/A
Haughton (2018) [42]	race/ethnicity	White participants, African American, Latinos, Asian, Native American, Hispanic	community engagement, community based participatory research, research partnerships, peer to peer sampling; the use of technology	N/A	N/A
Griffin (2021) [54]	race, ethnicity	White, African American/black, Asian/Pacific Islander, Native American, or other/unknown race.	N/A	N/A	N/A
Glover (2015) [25]	Indigenous people	N/A	Common factors that were seen as facilitators included partnership and	N/A	N/A

			relationship building (n = 25), culturally appropriate study design (n = 23), employing Indigenous staff (n = 20), targeted recruitment techniques (n = 21), and appropriate study materials (n = 20) (		
Franzen (2021) [66]	race and ethnicity	Whites, Asian, Black/African American, Latino	N/A	N/A	N/A
Fletcher (2022) [67]	Race and ethnicity	White, Black, Hispanic, Asian, and Other	One recent qualitative study conducted interviews with research staff at five U.S. cancer centers about perceived impediments to minority recruitment in clinical trials. A common theme that emerged was the need for improved training of research personnel at all levels (from study recruiters to principal investigators) on cultural awareness, patient-centered communication for varying levels of health literacy, and trust-building community engagement	N/A	N/A
Falasinnu (2018) [68]	Race and ethnicity	Whites, Blacks, Hispanics, Asians, Native Americans	N/A	N/A	N/A

Eichel (2021) [69]	race and ethnicity	White/European, Black/African American, Asian, Native/Aboriginal, Multiracial, Native Hawaiian/ Pacific Islander, Hispanic/Latinx	N/A	N/A	N/A
Cwalina (2022) [29]	race and ethnicity	White, Black, Hispanic, Asian, and other.	N/A	N/A	N/A
Cui (2015) [45]	race and ethnicity	race and ethnicity - Hispanic, White, Asian, Black, other	Presentations, flyers, brochures, posters, media advertisements, phone calls, and word-of-mouth	Culturally appropriate intervention activities<br><br>incentive - grocery gift cards, gifts, cash, food, recipe books, and exercise equipment, building staff- participant relationships, and regular communication with participants, such as thank-you notes, postcards, or project newsletters project bond,<br><br>participant convenience – e.g., transportation support to and from intervention activities or data collection, make-up sessions for missed intervention sessions, upcoming event reminders, childcare services, and optional days or home visits for data collection<br><br>Participant tracking - home visits for data collection.	N/A
Chen (2022) [51]	race and ethnicity	race and ethnicity - White, non-white	N/A	N/A	N/A
Charrow (2017) [9]	Race and ethnicity	White, Hispanic, Asian, African American	N/A	N/A	N/A
Carroll (2011) [40]	ethnicity	African American, Hispanic, Asian	Targeted advertisements, targeted mailings and/or telephone calls via use of phone lists from	Retention methods were described by 28 of the38 studies;<br>multiple contacts with participants during the study period; providing financial or other	N/A

			organizations, direct neighborhood linking recruitment to a cultural tradition, carefully planning culturally tailored, interactive pre-intervention/informational sessions<br><br>Research team members ethnically and/or linguistically matched to the study population collaboratively working with the partnering community organization and/or community advisory board	incentives, transportation, or child care were also commonly mentioned;<br><br>Caring attitude towards participants, demonstrate cultural sensitivity and respect for participants' life situations, meet regularly with community advisors and study staff for problem solving and be flexible or accommodating to location and timing of study visits as much as possible; having bicultural, bilingual, and/or ethnically matched study team members	
Canevelli (2019) [28]	race	White, Asian, Blacks, Hispanics -	N/A	N/A	N/A
Burnette (2022) [46]	race/ethnicity;	White, Asian, Hispanic, African American, Caucasian, Hispanic American	Clinical referrals and advertisements were the most common recruitment methods cited, but further details were rarely available.<br><br>Providing sufficient detail about recruitment is not only important for reproducibility but also to enhance understanding of how recruitment affects reach and inclusion.	N/A	N/A
Begic (2019) [52]	race/ethnicity, country of birth	White/Caucasian	N/A	N/A	N/A

Acton (2022) [77]	Race and ethnicity	White, Black, Asian, Hispanic	N/A	N/A	N/A
Hirano (2021) [53]	race and ethnicity	Black, Asian, Hispanic, White, Black, Asian, Hispanic	N/A	N/A	N/A
Orkin (2021) [26]	race/ethnicity	not specified	N/A	N/A	N/A
Riccioni (2023) [79]	Race/ethnicity	Predominantly Whites. Only 11 RCTs reported the per- centage for each racial group (American and Pacific Is- landers, Asian, Black, Hispanic, and White individuals)	N/A	N/A	N/A
Alvarez (2024) [80]	race and ethnicity	Whites, Black or African Americans, Asians, American Indians, and Pacific Islanders.	N/A	N/A	N/A
Buffenstein (2023) [83]	race and ethnicity	Hispanics, American Indians and Alaskan Natives, Asians, Whites, Native Hawaiians, Pacific Islanders, (Blacks and multiracial participants	N/A	N/A	N/A
De Jesús-Romero (2024) [71]	Race, ethnicity	White, Asian, Black	N/A	N/A	N/A
Delma (2023) [72]	Race, ethnicity	White, Black, Asian, Hispanic/Latino, Hawaiian/Pacific Islander, American Indian	N/A	N/A	N/A
Gala (2023)[73]	Race, ethnicity	White, Black, Hispanic, Other	N/A	N/A	N/A
Maria Guzmán (2023) [74]	Race, ethnicity	White, Black, Asian, American/Pacific, Islander, American Indian/Alaska Native	N/A	N/A	N/A
Issa (2023) [75]	Race, ethnicity	White, Black or African American, American Indian or Alaska Native was reported twice, and Native Hawaiian or other Pacific Islander	N/A	N/A	No

Kotlier (2024) [30]	Race, ethnicity	Not specified	N/A	N/A	N/A
Kou (2024) [34]	Race/ethnicity	Not specified – across different countries and cultures	N/A	N/A	N/A
Long (2024) [81]	Race/ethnicity	White, Asian Black, Hispanic, other, and American Indian/Alaska Native or Native Hawaiian/Pacific Islander.	N/A	N/A	Yes
Pathiyil (2023) [31]	Race, ethnicity	White, Asian, Black, and Pacific Islander.	N/A	N/A	N/A
Patki (2023) [32]	Race, ethnicity	White, Asian and Black	N/A	N/A	N/A
Pelton (2024) [84]	Race, ethnicity	White, Black, Asian, Hispanic/Latino	N/A	N/A	N/A
Riaz (2023) [85]	Race, ethnicity	White, Black, Hispanic, American Indian/Alaska Native, Asian/Pacific Islander	N/A	N/A	Yes
Steventon (2024) [76]	Race, ethnicity	Caucasian, East Asian, Black/African American, Hispanic or Latino, American Indian or Alaska Native, Native Hawaiian or Pacific Islander and Hispanic	N/A	N/A	N/A
Xiao (2024) [78]	Race, ethnicity	Hispanic, Latino, Black, Asian, Native Hawaiian or Other Pacific Islander, American Indian or Alaska Native	N/A	N/A	N/A
Talaski (2024) [35]	Race	Not specified	N/A	N/A	N/A

*N/A = not applicable
