# Supplementary material for: Recruitment, retention and reporting of variables related to ethnic diversity in randomised controlled trials: an umbrella review
Source: BMJ Open. 2024 Aug 9;14(8):e084889. doi: 10.1136/bmjopen-2024-084889 (PMC11340254; doi:10.1136/bmjopen-2024-084889)
Supplement: Supplementary file 3 [file bmjopen-2024-084889.reviewer_comments.pdf]

PEER REVIEW HISTORY

BMJ Open publishes all reviews undertaken for accepted manuscripts. Reviewers are asked to complete a checklist review form (**http://bmjopen.bmj.com/site/about/resources/checklist.pdf**) and are provided with free text boxes to elaborate on their assessment. These free text comments are reproduced below.

ARTICLE DETAILS

**TITLE (PROVISIONAL)**	Recruitment, retention, and reporting of variables related to ethnic diversity in randomised controlled trials: An umbrella review
**AUTHORS**	Owusu-Addo, Ebenezer; Mensah, Deborah B; Orkin, Aaron; Chan, An-Wen; Welch, Vivian A.; Treweek, Shaun; Green, Heidi; Feldman, Peter; Ghersi, Davina; Brijnath, Bianca; Group, RECONSIDER Extension

VERSION 1 – REVIEW

**REVIEWER**	Kanellakis, Nikolaos I.<br>University of Oxford, Nuffield Department of Medicine
**REVIEW RETURNED**	14-Feb-2024

**GENERAL COMMENTS**	The pandemic of COVID-19 showed the importance of health equity and the barriers imposed by health disparities. The scientific community has acknowledged the significance of clarity when reporting RCTs.<br>The authors show that ethnic and other social variables are underreported. While more than 95% of the studies report gender in the demographics about half would fail to include ethnic details.<br>The authors recognise two limitations of the current study which are both sensible.<br>This is a very interesting study which could inform RCT conduct.

**REVIEWER**	Schick , Melissa<br>Yale School of Medicine
**REVIEW RETURNED**	20-Apr-2024

**GENERAL COMMENTS**	This manuscript presents an umbrella review examining the extent to which variables related to ethnic diversity have been the focus of previous systematic reviews, inclusive of reporting on participant ethnicity, summarizing considerations for recruitment and retention of members of minoritized ethnic groups, and analyses that consider ethnicity. This is an important endeavor that suggests that science as a whole needs to improve efforts in these areas, rather than potential field specific effects (as have been suggested by previous reviews). Overall, I found the manuscript compelling and well-written. I had just a few suggestions for improvement:<br><br>1. The title does not fully capture the scope of this review – given that it only focused on reporting of ethnic diversity, I was surprised to find that recruitment, retention, and analysis were also a focus.<br>2. The review's timeframe was unclear. The abstract says that articles were included if they were published any time before 2022, and the limitations section notes that some of the literature drawn upon was 23 years old, but the methods section notes that reviews

	were only included if they came out between 2010 and 2022 and the PRISMA diagram notes that articles were excluded if they were published before 2010. If it is the case that this lower limit was imposed, a justification for why should be provided.<br>3. Relatedly, the review should be updated for manuscripts that came out, at the least, in late 2022 and 2023 (given that the search was conducted in early-to-mid 2022 – nearly two years ago).<br>4. It would be helpful to include some operationalization and examples of what would be considered recruitment, reporting, retention, and analysis. Until I reached the results, I was confused by what the distinction would be between recruitment and reporting.<br>5. I don't believe that the groups listed under Ethnic/racial categories in "Additional file 4" – I see a few articles for which the table is missing some groups that are included in the respective articles. There are also some cells in this table that are blank.<br>6. It is a bit hard to tell what is being represented in Figures 2 and 3 – is it the number of reviews that suggested each item on the X-axes as a recruitment and retention strategy to improve representation?

VERSION 1 – AUTHOR RESPONSE

**Reviewer 1**	
1. The pandemic of COVID-19 showed the importance of health equity and the barriers imposed by health disparities. The scientific community has acknowledged the significance of clarity when reporting RCTs.<br>The authors show that ethnic and other social variables are underreported. While more than 95% of the studies report gender in the demographics about half would fail to include ethnic details. The authors recognise two limitations of the current study which are both sensible.<br>This is a very interesting study which could inform RCT conduct.	1. Thank you for acknowledging the value and the contribution of this review to improving future trials.
**Reviewer 2**	
1. This manuscript presents an umbrella review examining the extent to which variables related to ethnic diversity have been the focus of previous systematic reviews, inclusive of reporting on participant ethnicity, summarizing considerations for recruitment and retention of members of minoritized ethnic groups, and analyses that consider ethnicity. This is an important endeavor that suggests that science as a whole needs to improve efforts in these areas, rather than potential field specific effects (as have been suggested by previous reviews). Overall, I found	1. Thank you for acknowledging the value and the contribution of this review to improving future trials.<br><br>2. We have revised the title to capture the full breadth of the review as follows:<br>***Recruitment, retention and reporting of variables related to ethnic diversity in randomised controlled trials: An umbrella review*.**<br><br>3. Please see response to point 1 of the same comment from the Editor.

the manuscript compelling and well-written. I had just a few suggestions for improvement:<br><br>2. The title does not fully capture the scope of this review – given that it only focused on reporting of ethnic diversity, I was surprised to find that recruitment, retention, and analysis were also a focus.<br><br>3. The review's timeframe was unclear. The abstract says that articles were included if they were published any time before 2022, and the limitations section notes that some of the literature drawn upon was 23 years old, but the methods section notes that reviews were only included if they came out between 2010 and 2022 and the PRISMA diagram notes that articles were excluded if they were published before 2010. If it is the case that this lower limit was imposed, a justification for why should be provided. Relatedly, the review should be updated for manuscripts that came out, at the least, in late 2022 and 2023 (given that the search was conducted in early-to-mid 2022 – nearly two years ago).<br><br>4. It would be helpful to include some operationalization and examples of what would be considered recruitment, reporting, retention, and analysis. Until I reached the results, I was confused by what the distinction would be between recruitment and reporting.<br><br>5. I don't believe that the groups listed under Ethnic/racial categories in "Additional file 4" – I see a few articles for which the table is missing some groups that are included in the respective articles. There are also some cells in this table that are blank.<br><br>6. It is a bit hard to tell what is being represented in Figures 2 and 3 – is it the number of reviews that suggested each item on the X-axes as a recruitment and retention strategy to improve representation?	We have updated the review to capture papers published until May 13, 2024. 19 new reviews were added increasing the number of included reviews from 43 to 62.<br>This, however, did not change the conclusions of the review as the additional papers reported similar findings to the previously included papers.<br><br>4. We have defined these in the section on inclusion criteria on page 5 as follows:<br><br>*Recruitment: defined as identifying or sourcing participants who may be eligible for the trial. We included review that described recruitment of participants and/or strategies used to enrol participants in trials.*<br><br>*Retention: defined as keeping enrolled participants in a trial from discontinuing or dropping out. We included reviews that described strategies used to keep participants enrolled in trials.*<br><br>*Analysis: defined as analysis of ethnic diversity variables included in a trial. We included reviews that focused on an analysis of ethnic diversity variables in trials.*<br><br>*Reporting: we included reviews that focused on reporting or representation of ethnic diversity in trials.*<br>5. In additional file 4, we provide information on ethnic/racial categories as reported by the included reviews. In instances where these are missing from the articles, we are handicapped in providing them. Also, because the review focuses on reporting, recruitment, retention, and analysis of ethnicity in trials, we provide the relevant information that are applicable to a particular review. For instance, if an included review focuses on recruitment (e.g., Masood 2019), reporting and analysis become not applicable, that is why we have provided N/A in those instances. There were also instances where a review will not provide details of the ethnic groups.<br>Heller's (2014) review focused on Strategies addressing barriers to clinical trial enrollment of underrepresented populations. We have provided relevant information on ethnic diversity in this review.<br><br>6. We have deleted these figures as they add little value.

VERSION 2 – REVIEW

**REVIEWER**	Schick , Melissa<br>Yale School of Medicine
**REVIEW RETURNED**	30-May-2024

**GENERAL COMMENTS**	This is a revised manuscript presenting the results of an umbrella review examining the extent to which recruitment and retention of ethnically minoritized individuals, and analysis and reporting of variables related to ethnic diversity have been the focus of previous systematic reviews. I continue to believe that this is an important topic, and that this umbrella review will be of great interest to readers. I appreciate the revision made to the title (which I believe now more fully summarizes the scope of this review) and the effort to update the review through 2024 (which is certainly not an easy undertaking). I had just a few points that I do not believe were addressed in this revision:<br><br>1. I had previously noted that some justification should be provided for limiting the search to articles published since 2010. I do not see this change reflected, but still think it would be useful to understand the rationale for including only more recent reviews.<br>2. I had also previously noted that there were some articles for which Additional file 4 was missing some groups that were included in respective articles. The authors responded that they included any information reported by each review. I apologize for any lack of clarity – I meant to indicate that I see a few instances where information about an ethnic group was included in the review, but it is not represented in the table (e.g., the cell "Ethnic/racial categories" for Schick, 2020 lists White, African American, Asian, but the review itself includes more categories than this – White, Black/African American, Hispanic/Latinx, Asian, American Indian/Alaska Native, Multiracial). Note that Schick, 2020 is one example – there are others that I know of that are incorrect, so the entries may need to be double checked.<br>3. In Additional file 4, the need to have some cells listed N/A also makes sense, but there are some cells that are blank (do not have any text, or N/A included; e.g., Kwiatkowski, 2013 has a blank cell for "Retention strategies"). What do these blanks represent?

VERSION 2 – AUTHOR RESPONSE

**Reviewer 1**	
1. This is a revised manuscript presenting the results of an umbrella review examining the extent to which recruitment and retention of ethnically minoritized individuals, and analysis and reporting of variables related to ethnic diversity have been the focus of previous systematic reviews. I continue to believe that this is an important topic,	1 Thank you appreciating the value addition of this umbrella review.

and that this umbrella review will be of great interest to readers. I appreciate the revision made to the title (which I believe now more fully summarizes the scope of this review) and the effort to update the review through 2024 (which is certainly not an easy undertaking). I had just a few points that I do not believe were addressed in this revision:	
2. I had previously noted that some justification should be provided for limiting the search to articles published since 2010. I do not see this change reflected, but still think it would be useful to understand the rationale for including only more recent reviews.	2. Our sincere apologies for failing to address this previews comment. It was an oversight.<br><br>We have provided the following information, which justifies the decision to include papers published between 2010 and 2024.<br><br>This umbrella review was conducted to inform the development of guidelines on inclusion and reporting of ethnicity in trials (RECONSIDER Extension). Therefore, to ensure that the guideline is up to date, the search was limited to 14 years and included reviews published during the timeframe of January 1, 2010 − May 13, 2024.
3. I had also previously noted that there were some articles for which Additional file 4 was missing some groups that were included in respective articles. The authors responded that they included any information reported by each review. I apologize for any lack of clarity – I meant to indicate that I see a few instances where information about an ethnic group was included in the review, but it is not represented in the table (e.g., the cell “Ethnic/racial categories” for Schick, 2020 lists White, African American, Asian, but the review itself includes more categories than this – White, Black/African American, Hispanic/Latinx, Asian, American Indian/Alaska Native, Multiracial). Note that Schick, 2020 is one example – there are others that I know of that are incorrect, so the entries may need to be double checked.	3. Thank you for clarifying your previous point. We have double checked the included reviews and have updated this section. All missing information relating to race/ethnicity have been provided and highlighted in yellow text.
4. In Additional file 4, the need to have some cells listed N/A also makes sense, but there are some cells that are blank (do not have any text, or N/A included, e.g., Kwiatkowski, 2013 has a blank cell for “Retention strategies”). What do these blanks	

represent?	4. This has been corrected. Thank you.
Reviewer 2	
No comments	
